# A pedicled buccal periosteal flap for the closure of oro-antral fistula

**DOI:** 10.1186/s12903-024-04217-6

**Published:** 2024-04-10

**Authors:** Marwa T. Ibrahim, Eslam A. Gharieb, Mona S. Sheta

**Affiliations:** 1 Department of Oral and Maxillofacial Surgery, Faculty of Dentistry, Tanta University, El-Giesh St, Tanta, Gharbia Egypt; 2 Department of Oral and Maxillofacial Surgery, Tanta University, El-Giesh, Tanta, Egypt

**Keywords:** Pedicled buccal periosteal flap, Oroantral fistula, Maxillary sinus

## Abstract

**Background:**

An oroantral fistula is a communication between the maxillary antrum and oral cavity. This pathological communication is formed mainly due to dental extraction of maxillary premolars and molars. Adequate management should include closing the oroantral fistula and eliminating sinus infections to prevent recurrence and sinusitis.

**Purpose:**

This study aimed to evaluate the effectiveness of using the pedicled buccal periosteal flap for closing an oroantral fistula without changing the native intraoral structure.

**Patients & Methods:**

Patients with oroantral fistulas were included in this study. The patients were examined clinically by Valsalva test and cheek-blowing test, the hole was probed, and the extent of the underlying bone defect was determined radiographically using computed tomography preoperatively. All patients underwent surgical closure of oroantral fistula using a pedicled buccal periosteal flap.

**Results:**

All 10 patients obtained satisfactory results with marked improvement in the function of the maxillary sinus and complete healing of oroantral fistula with no recurrence except in Case No. 5, who had a recurrence of the oroantral fistula, also there was no statistically significant difference between the vestibular depth preoperatively and postoperatively.

**Conclusion:**

A pedicled buccal periosteal flap is a novel technique for oroantral fistula closure as it preserves vestibular depth with a tension-free closure flap and harbors the advantages of the regenerative potential of the periosteum.

**Registration date:**

14/8/2023

**Registration number:**

NCT05987943

## Background

An oroantral fistula (OAF) is characterized as a channel bordered by epithelium that may be filled by granulation tissue or sinus membrane polyposis. Trauma, tooth infection, osteomyelitis, radiation therapy, bone preparation for the placement of a dental implant, or excision of maxillary pathology can all induce OAF. Because of the closeness of the premolar apices and molars to the maxillary sinus floor, the extraction of upper posterior teeth is the most common cause of OAF [1–3].

The majority of oroantral communications of more than 5 mm that persist for more than 3 weeks will epithelialize and become persistent chronic oroantral fistulas, which are indicated for surgical repair [4].

Borgonovo et al. [5], in 2012, advised using the buccal flap to close moderate-sized oroantral fistulas, provided that they are not too posteriorly located, the palatal flap for fistulas in the premolar teeth region, and the buccal flap coupled with buccal fat pad shift for fistulas in the third molar region.

To surgically repair the oroantral fistula, a variety of soft tissue flaps are utilized, including local or distant flaps, that are randomly or axially based and have a single or double-layered closure. The buccal sliding flap and the pedicled buccal fat pad graft are examples of local flaps. The main drawbacks of these procedures include the loss of vestibular depth, which may demand a secondary vestibuloplasty for patients who wore dentures, the reduction of keratinized gingiva height, closure under strain, and therefore increased susceptibility to wound dehiscence [6, 7]. 

Technical issues associated with palatal rotation flaps have included the greater palatine artery kinking and bunching the flap along its axis of rotation, and the anterior donor location has a painful bare bone. While the palatal island flap is more flexible for side rotation and has less exposed raw bone, the vascular pedicle is more prone to stress [8]. 

The tongue flap, temporalis flap, temporalis myofascial flap, and microvascular transfers are the most helpful remote soft tissue flaps. They offer a superior bulk of tissue for closing large defects. However, these approaches necessitate general anesthetic operations alongside high morbidity, hemorrhage, infection, and flap failure [9]. 

Nevertheless, all approaches change the native oral architecture and can cause severe postoperative morbidity. The pedicled buccal periosteal flap is introduced in this article as a safe and simple approach for closing OAF without changing the original intraoral architecture. Because the periosteum includes desirable stem and progenitor cells as well as significant vascular-proliferative and neuro-trophic capabilities, its regeneration potential is considerable. For the proper use of this capacity, many surgical and/or tissue engineering procedures have been suggested [10]. 

The purpose of this study was to evaluate the effectiveness of using the pedicled buccal periosteal flap for closing an oroantral fistula, the investigators hypothesize that using this technique improves the function of the maxillary sinus and preserves the vestibular depth. The specific aim of the study was the surgical closure of the oroantral fistula using the pedicled buccal periosteal flap and measuring its effect on the vestibular depth.

## Materials and methods

### Study design

The investigators designed and implemented an uncontrolled clinical trial and the patients with OAF were recruited from the Oral and Maxillofacial Surgery Department Outpatient Clinic, Faculty of Dentistry, Tanta University, between September 2022 and May 2023.

The research for this study received approval from Tanta University’s Faculty of Dentistry Research Ethics Committee under code (#R-OS-9-22-6). Following the standards for human research approved by the Research Ethics Committee of the Faculty of Dentistry, Tanta University which follows the ethical guidelines outlined in the 1964 Helsinki Declaration and its subsequent revisions, the patients’ objective for participating in the study was described to them, and their informed consent was obtained prior starting treatment. NCT05987943 is the approved clinical trial number.

### Eligibility criteria

**Fig. 1:**
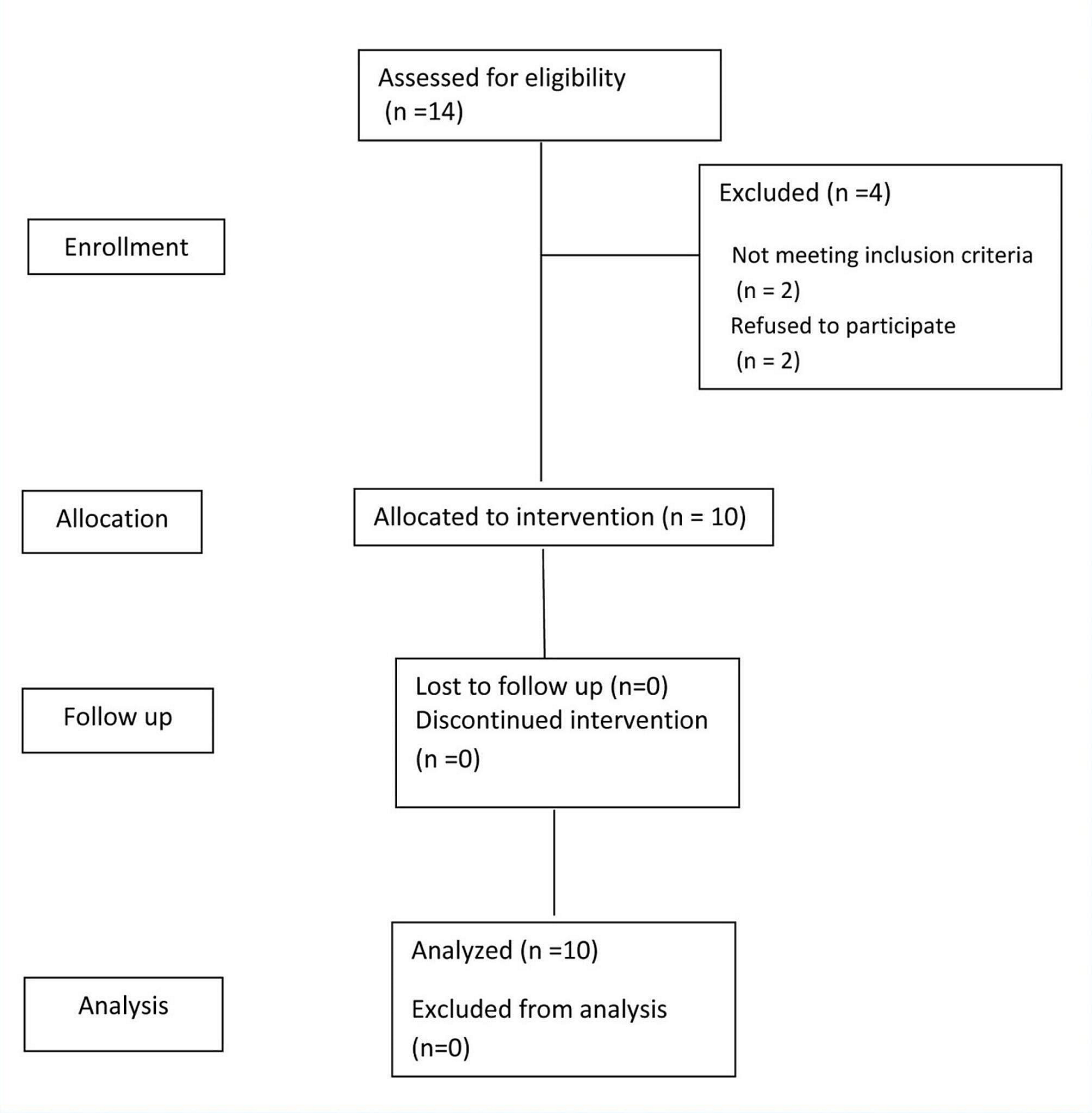
CONSORT flow chart

Patients were selected according to the following main inclusion and exclusion criteria. The main inclusion criteria were patients complaining of nasal regurgitation of fluids, unilateral nasal discharge, changed nasal resonance, foul taste in the mouth, difficulty sucking via a straw and whistling sound when speaking and pain in the malar area. Patients with OAF less than 2 mm, debilitating diseases and not willing to participate in the study were excluded (Fig. 1).

### Sample size calculation

The sample size was calculated using G*Power software and sample size calculations Version 3.1.9.4. This power analysis used the difference in vestibular depth as the primary outcome. The effect sizes d = 1.33 was calculated based upon the results of Kumar et al. [11] and the estimated mean difference between the two groups = 0.18. Using alpha (α) level of (5%) and beta (β) level of (20%), that is, power = 80%; the minimum estimated sample size was a total of 7 subjects. The sample size will be increased to a total of 10 subjects to compensate for a dropout rate of about 20%.

### Preoperative evaluation

#### Chief complaint

Symptoms (e.g., nasal regurgitation of fluids, unilateral nasal discharge changed nasal resonance, foul taste in the mouth, difficulty sucking via straw and whistling sound when speaking) are the main complaints. Pain in the malar area is possible.

#### Clinical examination

Valsalva test, cheek-blowing test, perforation investigation with probing and vestibular depth measured from gingival margin to bottom of the vestibule.

#### Radiographical examination

**Fig. 2:**
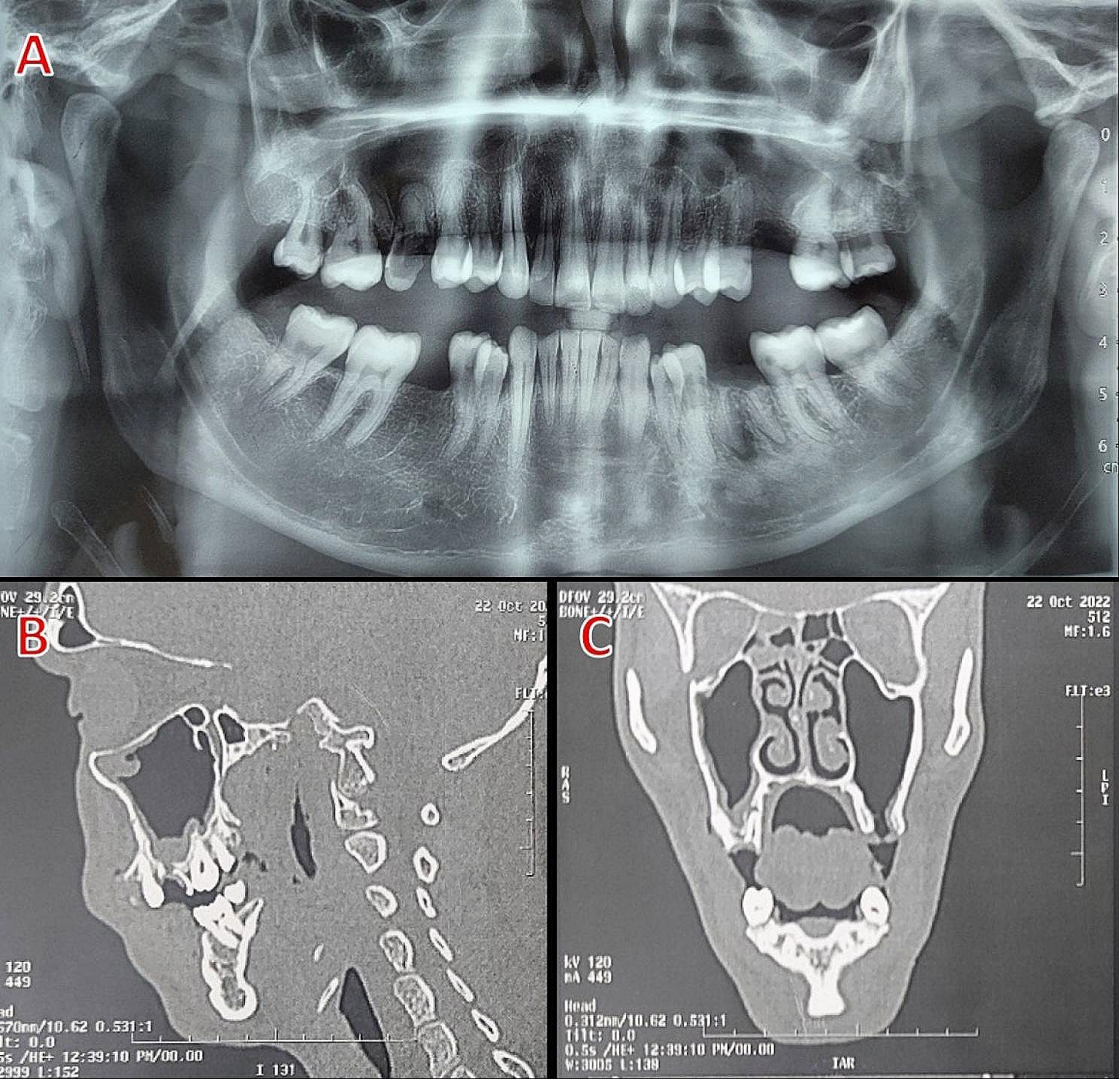
(**A**) Panoramic X-ray film demonstrating an oroantral fistula associated with the extracted upper left first molar. (**B**&**C**) A sagittal and coronal CT scan of the extraction site reveals an oroantral fistula, Case No. 2

Panoramic radiograph to determine the location of the fistula as well as the existence and position of dental roots, implants, or any foreign bodies that may have become displaced within the maxillary sinus. Computed tomography (CT) scans were used to evaluate the magnitude of the underlying bone defect, exclude the occurrence of maxillary sinusitis, identify the existence of foreign bodies within the maxillary sinus cavity, and determine discontinuity of the maxillary sinus floor as shown in (Fig. 2).

### Preoperative management

The affected maxillary sinus should be irrigated with normal saline to eliminate infection through the fistulous orifice, followed by a betadine-containing solution diluted with normal saline. This process should be carried out until the lavage fluid is plain and without any inflammatory exudates.

### Surgical procedure

All patients were treated with the pedicled buccal periosteal flap procedure under local anesthesia (4% articaine and epinephrine 1:100.000) using maxillary block and vestibular infiltration. To reveal the underlying connective tissue, a circular supra-periosteal incision was made with a 15-scalpel to eradicate the epithelial tissue along the fistula boundary. Following the elimination of the epithelial fistula wall, the sinus cavity was thoroughly curettaged and rinsed with saline solution to eliminate diseased and necrotic tissue.

**Fig. 3:**
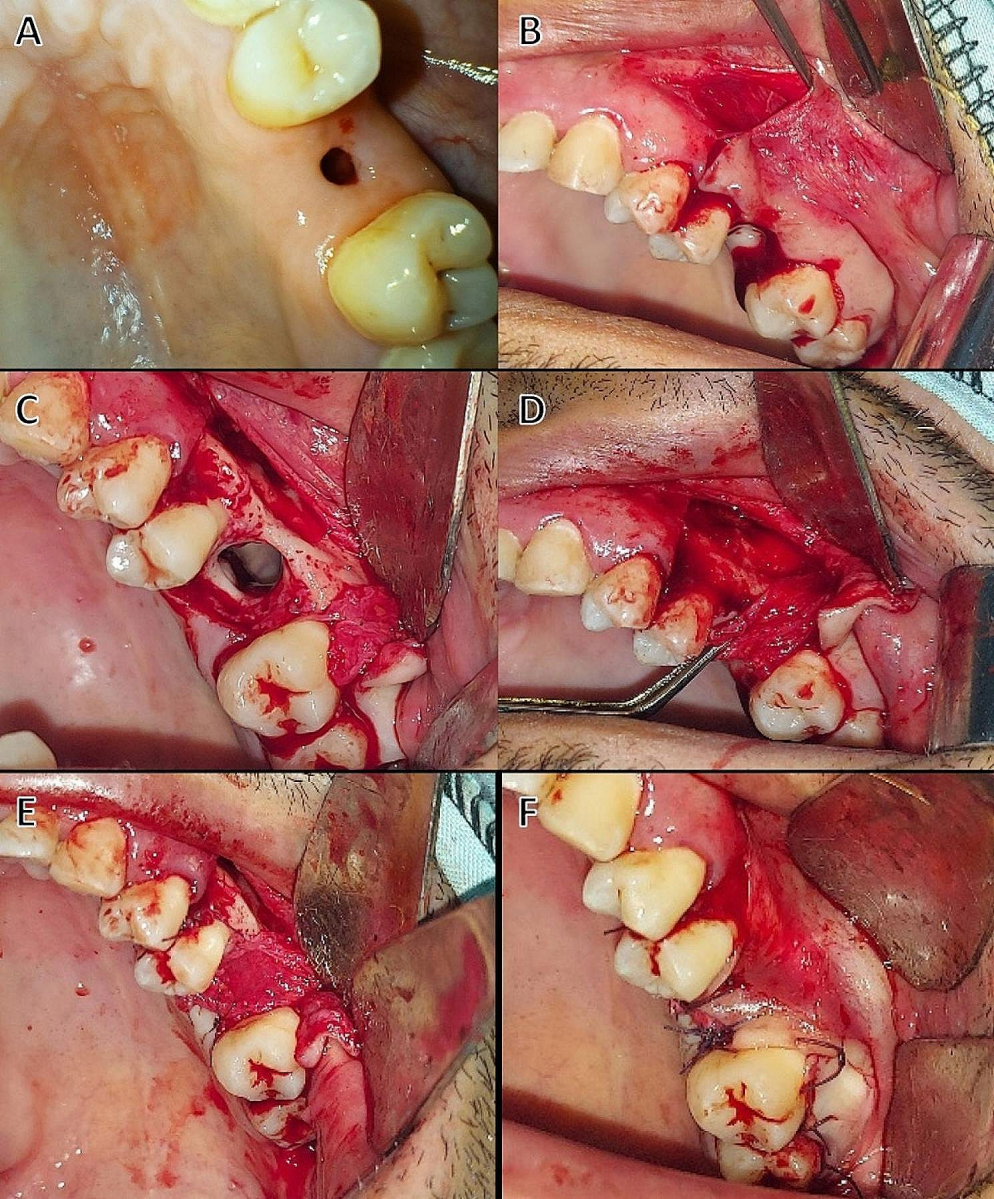
(**A**) Preoperative photograph of an oroantral fistula (**B**) incision and reflection of buccal mucosal flap, (**C**) bony defect related to the oroantral fistula of upper left first molar, (**D**) reflection of periosteal flap from the underlying bone, (**E**) suturing of the periosteal flap over the oroantral fistula, (**F**) suturing of the buccal mucosa to the palatal tissue, Case No. 2

A crestal incision was made with a Number 15 Bard Parker blade, with an anterior oblique releasing incision to the fistula. The buccal mucoperiosteal flap was split horizontally into two layers above the mucogingival junction: the first layer was a deep periosteal layer which was dissected submucosal about a distance of one tooth from the fistula while the second layer was a superficial buccal mucosal layer. Sutures were used to stabilize the pedicled deep periosteal layer, which was separated and turned above the oroantral fistula at the bone level and sutured to the palatal tissue. The superficial layer of buccal mucosa was bluntly dissected and sutured to the palatal tissue. In all cases, immediate evaluation with the Valsalva maneuver and probing with a blunt object revealed the main water-tight closure of the fistula as shown in (Fig. 3).

### Postoperative management

Patients were advised to consume soft foods and avoid maneuvers such as sneezing with one’s mouth closed and nasal blowing that might raise the intrasinus pressure until healing occurred.

For two weeks, warm saline was used to keep the wound clean and the mouth was rinsed with 0.12% chlorhexidine digluconate mouthwash. To maintain antral orifice patent for drainage, all patients were given antibiotics, nonsteroidal anti-inflammatory drugs and nasal decongestants for at least 7 days.

### Postoperative evaluation

The patients were observed weekly for one month, and later after three months. OAF closure was examined for healing, inflammation, infection, and any recurrence. Pain levels were measured using a 0–10 visual analogue scale (VAS), with 0 indicating no pain and 10 indicating the most severe pain, and the vestibular depth was measured from the gingival margin to the bottom of the vestibule using a periodontal probe.

## Results

### Demographic data

Ten patients were included in this study (6 males, 4 females) with a mean age of 36 years (ranging from 22 to 49 years) and in all cases, the major reason for OAF development was dental extraction. Case No. 8 had one previous failed OAF closure trial prior to entrance to our department, whereas the other patients were experiencing their first.

The average size of a soft tissue fistula was 4.7 mm (ranging from 0 to 9 mm), while the average size of a bone defect was 8.4 mm (range 4–12 mm). Table 1 shows that the average duration of a fistula was 14.4 weeks with (range 0–48) weeks as case No. 9 was oroantral communication due to extraction of the upper left second molar with periapial granuloma, as shown in (Table 1).

**Table 1:** Demographic data and features of OAF

No	gender	Age (years)	Size of Fistula (mm)	Size of bony defect (mm)	Fistula duration (weeks)	Cause of OAF
1	male	41	7	10	14	Dental extraction
2	male	35	9	12	48	Dental extraction
3	female	22	3	4	10	Dental extraction
4	male	39	4	7	8	Dental extraction
5	male	34	6	10	12	Dental extraction
6	male	43	3	6	6	Dental extraction
7	female	49	7	10	11	Dental extraction
8	female	26	5	7	20	Recurrent OAF
9	male	33	0	11	0	Dental extraction
10	female	38	3	7	15	Dental extraction

#### Clinical assessment

##### 1-Pain

: The mean postoperative VAS pain level was 5.5 (ranging from 4 to 9) at the first- week postoperative follow-up, 2.5 (ranging from 1 to 6) at the second- week follow-up, 1 (ranging from 0 to 4) at the third week follow-up, and 0 at the fourth-week follow-up.

##### 2-Healing

**Fig. 4:**
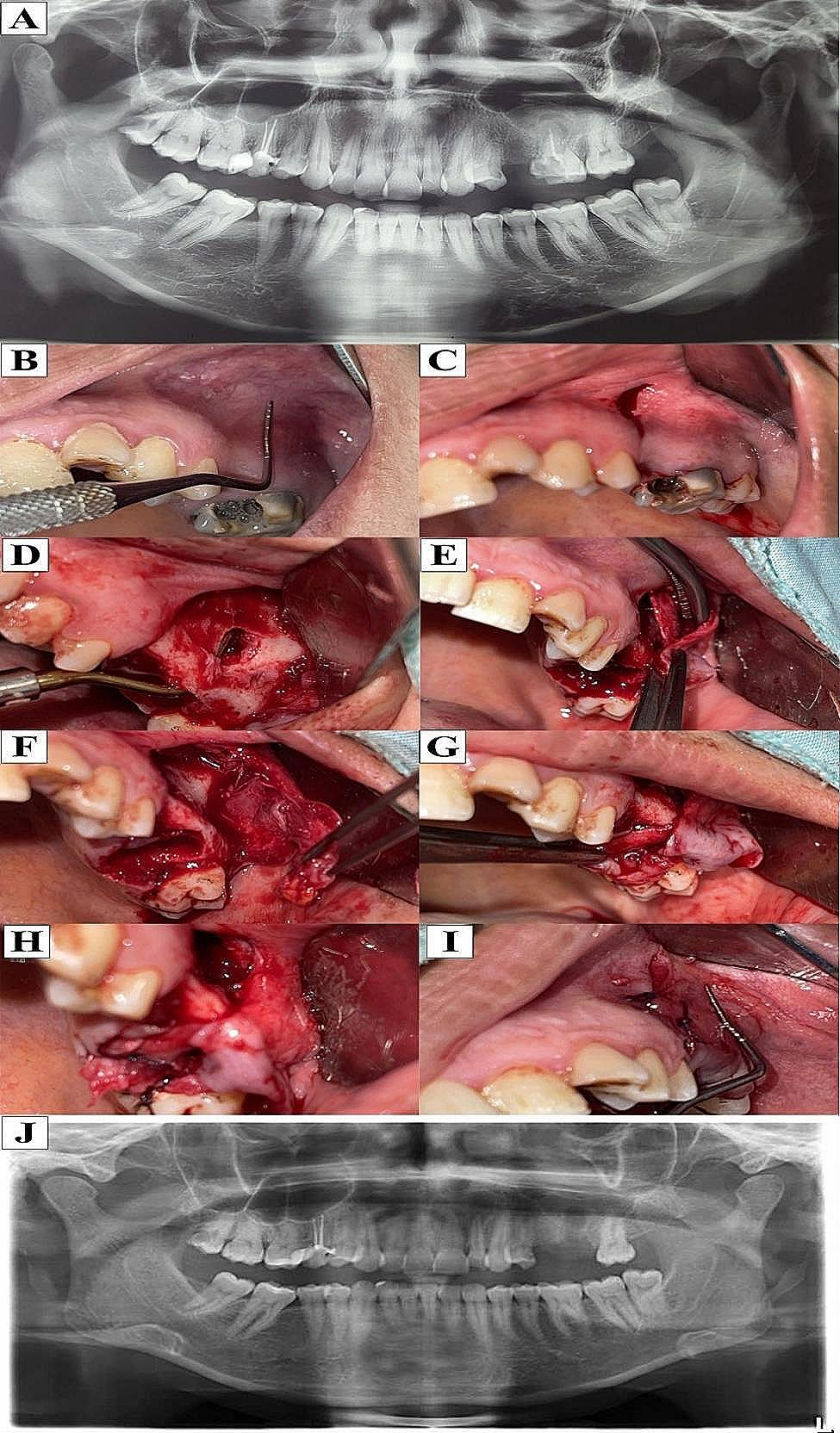
(**A**) Preoperative panoramic X-ray film (**B**) Preoperative photograph showing the vestibular depth of buccal sulcus before extraction of upper left first molar (**C**) Incision of buccal mucosal flap, (**D**) Reflection of the flap showing bony defect related to the oroantral fistula of upper first molar, (**E**, **F**,**G**) Dissection of periosteal flap from the overlying buccal mucosa, (**H**) Suturing of the periosteal flap over the oroantral fistula, (**I**) Suturing of the buccal mucosa to the palatal tissue with preservation of vestibular depth (**J**) Postoperative panoramic X ray, Case No 9

**Fig. 5:**
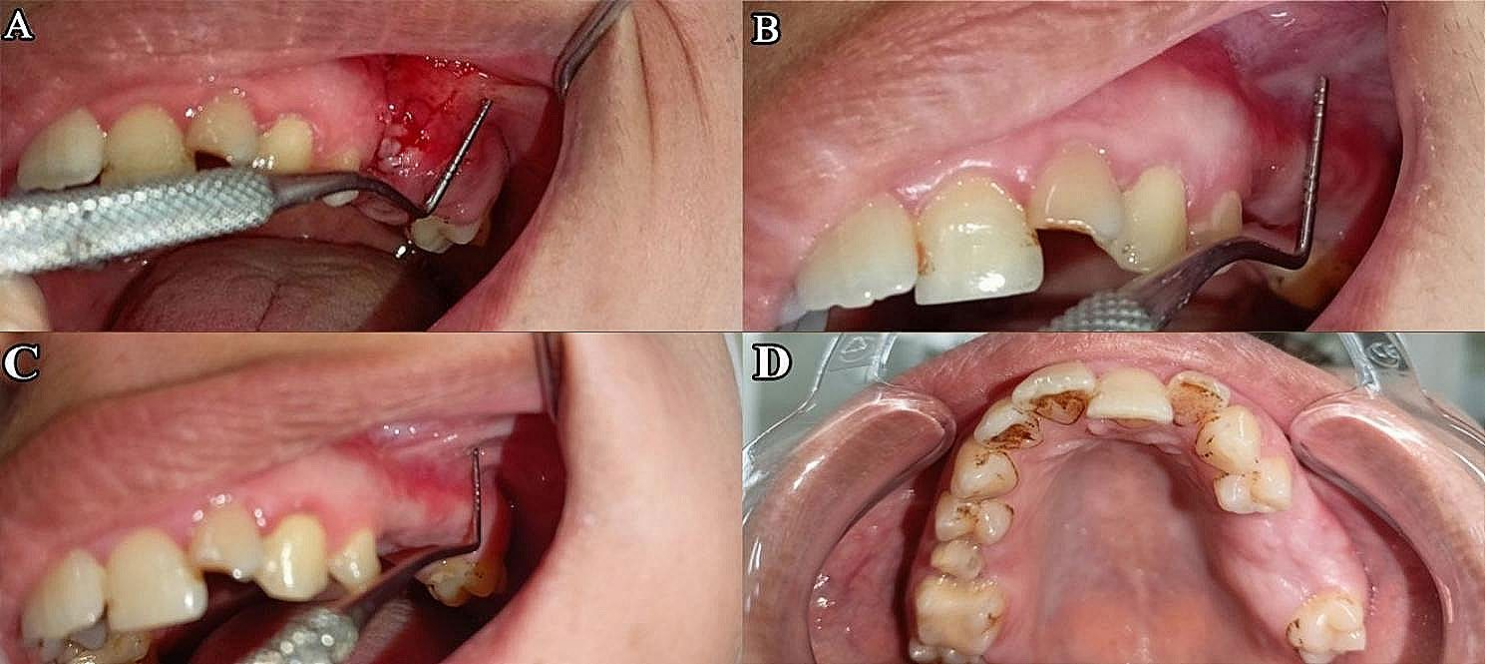
(**A**) Two weeks postoperatively showing slight inflammation at the surgical site (**B**) One month postoperatively showing adequate healing of oroantral fistula with preservation of vestibular depth, (**C**) Three months postoperatively buccal view and (**D**) occlusal view showing preservation of vestibular depth, Case No 9

At the 1-month postoperative follow-up, all fistulas were entirely healed (90%) without signs of infection in the sinuses and no evidence of recurrence, except case No 5 (10%) who had a 3 mm residual postoperative fistula with no evident cause and had been repaired with a local flap, At the 3-month postoperative follow-up all fistula were entirely healed (100%) with total closure of the oroantral fistula, which could be verified through a negative Valsalva maneuver, as shown in (Figs. 4 and 5).

##### 3- The vestibular depth

The preoperative value of vestibular depth ranged from 7.3 mm to 8.7 mm with a mean of 7.99 and standard deviation (SD) of 0.137, while the postoperative value ranged from 7.1 mm to 8.1 mm with a mean of 7.81 and SD of 0.142. The difference between preoperative and postoperative was not statistically significant (*P*-value < 0.05).

## Discussion

Oroantral communications smaller than 2 mm in diameter may heal spontaneously; however, OAFs larger than 3 mm in diameter should be surgically closed due to the risk of maxillary sinus inflammation and infection [12]. The current investigation found that oroantral fistulas were larger than 4 mm in diameter, which is consistent with the findings of Punwutikorn et al. [2].

This study included ten patients with a mean age of 36 years. Our findings matched those of Guven [13], who discovered that oroantral fistula most usually occurs after the 3rd decade of life. Our study included 6 males and 4 females which coincided with the findings of other authors, Punwutikorn et al. [2] and Lin et al. [14] who stated that females are more susceptible to oroantral fistula because they have larger sinuses than men.

The difference in vestibular depth between preoperative and postoperative periods was statistically insignificant (*P* value 0.05), which was consistent with a study that used a modified inverted periosteal flap versus a buccal advancement flap technique for oroantral fistula repair and found that during the follow-up period, average vestibular height in Group 1 patients remained stable [11]. While several procedures for closing oroantral fistulas have been developed, including the Rehrmann buccal flap, the fundamental issue is the loss of buccal sulcus height. The Moczair flap, a variant of this technique in which the buccal pedicle is laterally shifted, decreases this loss, but it is only available to individuals who are edentulous surrounding the connection [15–18]. 

When compared to previous surgical methods used to close an OAF, such as the palatal pedicled flap, which might induce discomfort in the donor site due to bare bone, the patients in this research did not complain of any discomfort as there is no bared bone related to technique used except in case No 2 which may be related to over stretching of the stitches at the upper premolar area and it was healed by secondary intension in the follow up period [17].

At the 1-month postoperative follow-up, all fistulas were entirely healed (90%) without signs of infection in the sinuses and no evidence of recurrence, except case No 5 (10%) which had a 3 mm residual postoperative fistula and this may be due to inadequate excision of epithelialized margins, inadequate trimming of bony margins, presence of any existing sinus infection, the patient may neglect the post-operative instructions in the follow-up period and this matching with the findings of Khandelwal [19].

Our technique includes the reflection of a partial-thickness flap, keeping the periosteum on the bone from where it is reflected, and downward advanced and put across the oroantral fistula as the periosteum has been identified as a source of therapeutically relevant stem and progenitor cells because periosteal cells can differentiate into osteoblasts, chondroblasts, fibroblasts, adipocytes and skeletal myocytes [20]. It holds unique qualities of strength and stability that enable this layer to seal the bony defect. It also induces a progressive proliferation of attached gingiva and thickening of the overlying tissue. This regenerative character plays an important role in the formation of a thick and stable keratinized gingiva in the region of the repaired fistula [21]. 

Duhamel [22] was the first to explore the periosteum’s osteogenic capacity. He observed that disturbing the periosteum induces new bone to develop. Periosteal cells have also been demonstrated to produce vascular endothelial growth factor, which assists in angiogenesis and wound healing [23]. Furthermore, one of our technique’s distinctive advantages is that the cell scaffold is autologous and host-immune reactions to the more often utilized graft or alloplastic membrane are avoided, which frequently results in graft rejection [24].

## Conclusion

A pedicled buccal periosteal flap is a unique OAF closure method that offers several benefits. It maintains the vestibular height while providing actual tension-free closure and simple accessibility and management of the periosteal flap.

## Data Availability

All data are available from the corresponding author upon reasonable request.
